# MicroRNA-155 Controls Exosome Synthesis and Promotes Gemcitabine Resistance in Pancreatic Ductal Adenocarcinoma

**DOI:** 10.1038/srep42339

**Published:** 2017-02-15

**Authors:** Manabu Mikamori, Daisaku Yamada, Hidetoshi Eguchi, Shinichiro Hasegawa, Tomoya Kishimoto, Yoshito Tomimaru, Tadafumi Asaoka, Takehiro Noda, Hiroshi Wada, Koichi Kawamoto, Kunihito Gotoh, Yutaka Takeda, Masahiro Tanemura, Masaki Mori, Yuichiro Doki

**Affiliations:** 1 Department of Gastroenterological Surgery, Graduate School of Medicine, Osaka University, Yamadaoka 2-2, Suita, Osaka, 565-0871, Japan; 2 Department of Surgery, Kansai Rosai Hospital, Inabasou 3-1-69, Amagasaki, Hyogo, 660-8511, Japan; 3 Department of Surgery, Osaka Police Hospital, Tennoji-ku Kitayamacho 10-31, Osaka, 543-0035, Japan

## Abstract

The cancer drug gemcitabine (GEM) is a key drug for treating pancreatic ductal adenocarcinoma (PDAC), but PDAC cells develop chemoresistance after long-term administration. Since the tolerance was immediately spread to every PDAC tissue in a patient, it is assumed that some certain efficient mechanisms underlay in the development of chemoresistance. Changes in the levels of particular microRNAs or alterations in intercellular communication play a dominant role in chemoresistance development, and recent data also suggest that exosomes play an important role in this process. In this study, we revealed that the loop conferred chemoresistance in PDAC cells. The loop was as follows; 1, The long-term exposure of GEM increased miR-155 expression in PDAC cells. 2, The increase of miR-155 induced two different functions; exosome secretion and chemoresistance ability via facilitating the anti-apoptotic activity. 3, Exosome deliver the miR-155 into the other PDAC cells and induce the following function. The target therapy to miR-155 or the exosome secretion effectively attenuated the chemoresistance, and these results were validated with both clinical samples and *in vivo* experiments. This mechanism represents a novel therapeutic target in GEM treatment to PDAC.

Pancreatic ductal adenocarcinoma (PDAC), a lethal neoplasm with a 5-year survival rate of approximately 5%, is increasing worldwide^1^,^2^,^3^,^4^. Several challenges must be overcome to improve the prognosis of this devastating malignancy: a lack of early detection because of the propensity for early local invasion or distant metastasis; limited treatment options; and an insufficient understanding of PDAC biology. Surgical resection remains the only curative treatment option, but only 20% of patients with PDAC are adopted for curative resection. Recent reports suggest that adjuvant chemotherapy following curative surgery significantly prolongs the overall survival time after surgery, and this approach is being adopted as a standard strategy^5^,^6^. Gemcitabine (GEM) is a key drug for treating PDAC, and it is commonly used for adjuvant chemotherapy. In fact, in the last decade, most PDAC patients have been treated with adjuvant GEM chemotherapy. Nonetheless, adjuvant chemotherapy with GEM only extends overall survival by about 6 months, with patients showing a median survival time of 22.1–23.6 months; this is not satisfactory one^5^,^7^. Although the majority of PDAC is sensitive to GEM at first, GEM cannot control PDAC for very long^8^, suggesting that PDAC develops resistance to GEM after prolonged exposure.

Some reports indicate that cell-cell interactions via exosomes or alterations in microRNA (miR) levels may play important roles in resistance development^9^,^10^. Exosomes, which are small (30–100 nm) vesicles that are secreted by various cells, mediate intercellular communication by transferring biologically active factors to recipient cells. MicroRNAs, which are ~21-nt RNA sequences that act as regulatory molecules, can target conserved sites in the 3′-untranslated regions (UTRs) of 5–100 mRNAs, thereby repressing the translation of the target mRNA^11^. Since one type of microRNA can affect the translation of many target mRNAs, alterations in the level of a single miRNA species can affect multiple cellular pathways. Either increases in exosome synthesis or alterations in microRNA levels could induce GEM resistance to PDAC, but the relationships of such changes and the underlying mechanisms remain unclear. We therefore considered two hypotheses. The first hypothesis was that alterations in the levels of a single type of microRNA in response to long-term GEM exposure induce exosome synthesis. This increase in exosomes could then mediate interactions between cancer cells, and the resulting exchange of biologically active factors would lead to GEM resistance. The second hypothesis was that exosome secretion by GEM-resistant cells delivers microRNAs that induce GEM resistance in other cells.

Notably, the mechanisms underlying GEM resistance have both clinical and biological relevance. We investigated these mechanisms using a GEM-resistant PDAC cell line that was established by long-term exposure of PDAC cells to GEM. We then validated our findings in clinical samples (i.e. resected specimens) by analyzing the miRNA levels along with the corresponding information of patient who had adjuvant GEM chemotherapy or post-recurrence chemotherapy. Here we report that one microRNA, namely miR-155, was detected in a comprehensive transcriptome survey of GEM-resistant PDAC cells and that it controls exosome secretion, thereby inducing GEM resistance in PDAC. These findings were validated in clinical samples and in a murine xenograft model.

## Results

### miR-155 overexpression in GEM-resistant PDAC cells and in exosomes that lead to GEM resistance

**Figure 1:**
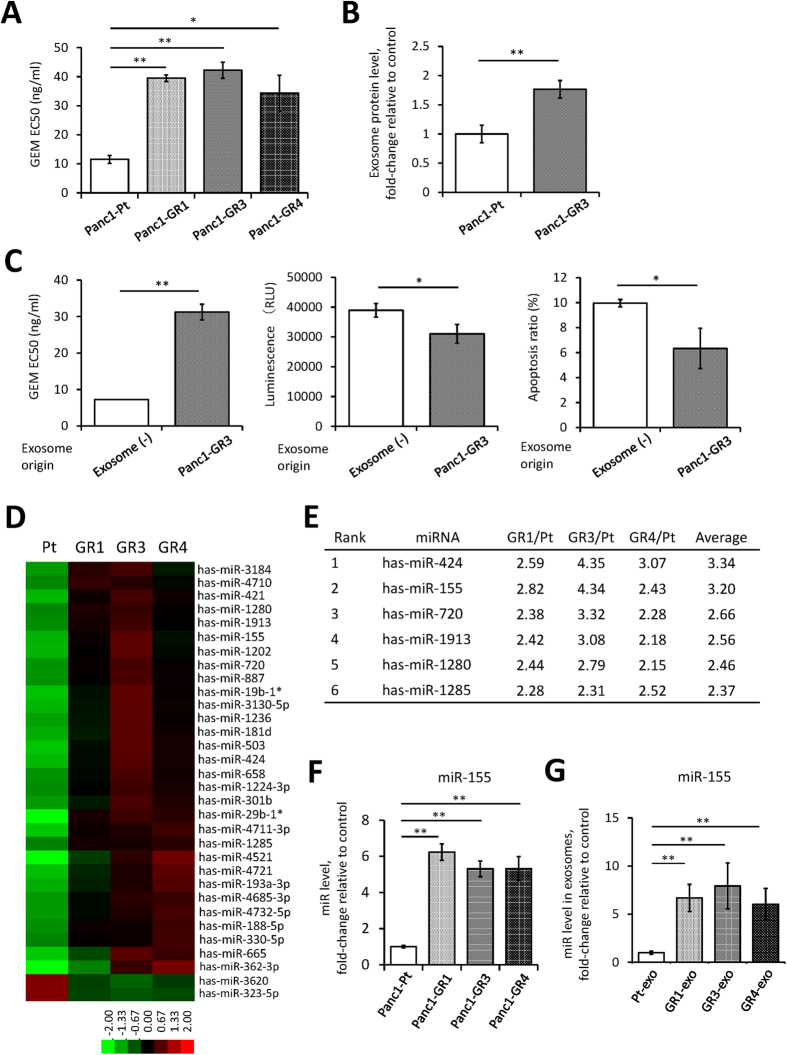
GEM-resistant PDAC cells show both upregulation of certain microRNAs and an increase in exosome synthesis. (**A**) The EC50 of GEM in each cell line as assessed by the MTT assay. GEM-resistant Panc1 (Panc1-GR) cells show stable GEM resistance compared to the parental Panc1 (Panc1-Pt) cells. (**B**) The protein levels in exosomes isolated from Panc1-Pt and Panc1-GR3 cells as assessed by the Bradford method. (**C**) Alterations in GEM sensitivity in Panc1-Pt cells with or without the addition of exosomes isolated from Panc1-GR3 cells. The EC50 was assessed by the MTT assay (left). The apoptosis assay was performed with 50 ng/ml GEM. Caspase-3/7 activity is represented as the level of luminescence (middle), and the ratio of apoptotic cells was determined using the Annexin V assay (right). (**D**) The heat map from the microarray analysis of miRNAs extracted from Panc1-Pt cells (Pt) or from Panc1-GR cells (GR1, GR3, GR4). miRNAs were selected for further analysis if the difference in their expression level in Panc1-GR cells was >2.0-fold or <0.5-fold relative to the expression in Panc1-Pt cells. (**E**) Ranking of the candidate miRNAs according to their fold-changes in expression. (**F** and **G**) The expression of miR-155 in each cell line (**F**) and in exosomes from each cell line (-exo) (**G**). Columns in (**A**–**C** and **F**) show values that are the averages of triplicate measurements, (**G**) show values that are the averages of ten times measurements; bars show SD values. Data are representative of three experiments. *P < 0.05; **P < 0.01.

Stable GEM-resistant Panc1 (Panc1-GR) cells (Fig. 1A) secreted more exosomes than the parental Panc1 (Panc1-Pt) cells (Fig. 1B). There was not the difference in the growth of these two cell lines in stable state. The increase of apoptosis ratio after GEM treatment was lower in GEM-resistant cell lines (Supplementary Fig. 1A and B). The exosomes from GEM-resistant cells elicited GEM resistance in GEM-sensitive cells (Fig. 1C), as reported previously^12^,^13^.

To identify candidate miRNAs that might be involved in the development of GEM resistance, we performed miRNA microarray studies. Of the 1719 miRNAs, 32 miRNA species showed marked changes in expression in Panc1-GR cells vs. Panc1-Pt cells. A total of 30 miRNAs showed at least a 2.0-fold increase, and 2 miRNAs showed a 0.5-fold decrease (Fig. 1D, Supplementary Table 1). We excluded 26 of these miRNAs because their expression was undetectable in Panc1-GR cells or because they were non-functional miRNAs; thus, there were 6 miRNAs that were considered candidates for inducing GEM resistance (Fig. 1E). Of these microRNAs, miR-155 showed the second highest alteration in expression, with an average 3.20-fold increase in expression in each of the Panc1-GR cells (Pan1-GR1, 3 and 4) vs. Panc1-Pt cells, and only this microRNA had been reported previously to be related to chemoresistance^14^,^15^. We therefore focused on miR-155 in further experiments after validating the upregulation of miR-155 both in cells and in exosomes using qRT-PCR (Fig. 1F and G).

### Patients receiving GEM treatment who show high miR-155 expression in their PDAC epithelial cancer cells show poor prognosis

**Figure 2:**
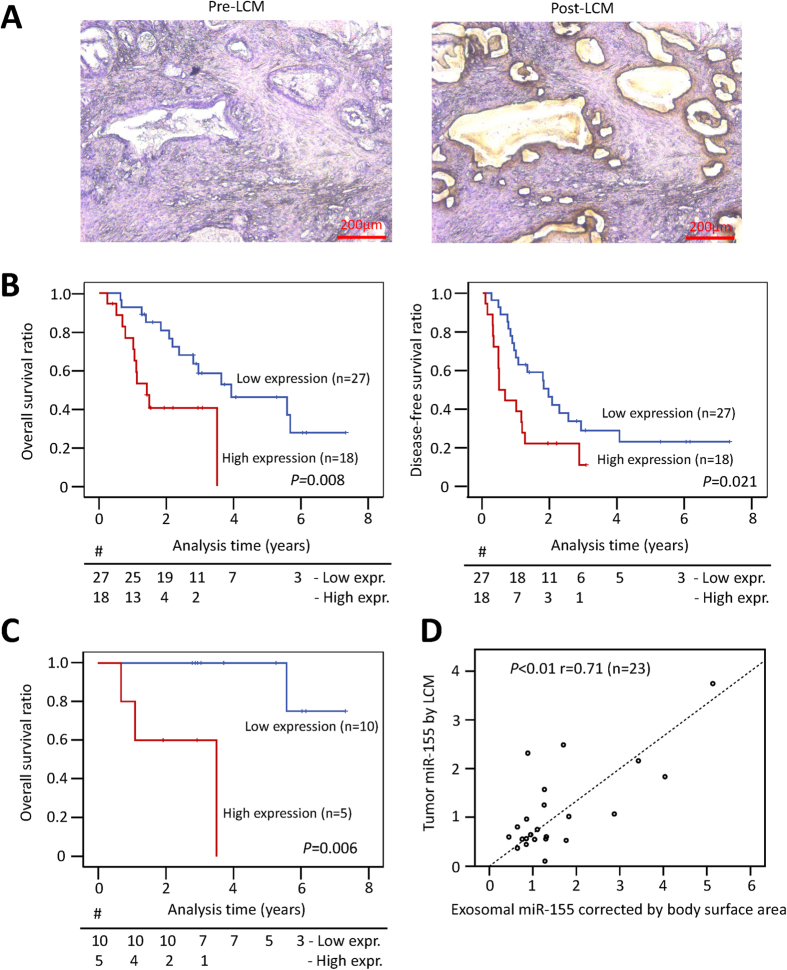
(**A**) Representative images pre- or post-laser capture microdissection (LCM) of resected human PDAC samples. Epithelial cancer ducts in PDAC tissue were captured and the microRNA was extracted. Scale bar, 200 μm. (**B**) Kaplan-Meier plots of overall survival (left) and disease-free survival (right) of 45 patients categorized according to high vs. low miR-155 expression (*P* value, log-rank test). (**C**) Of the 45 patients, 15 received adjuvant GEM therapy. Their overall survival rate is shown according to their miR-155 expression levels. (**D**) The correlation of miR-155 expression in resected tissue and the miR-155 levels in exosomes isolated from the corresponding patient’s plasma (*P* value, Pearson’s correlation).

To study the clinical significance of miR-155 expression, miR-155 levels were determined in PDAC epithelial cancer cell samples obtained by laser capture microdissection from 45 patients who received GEM chemotherapy after R0 resection (Fig. 2A). The expression level of miR-155 varied in the samples (Supplementary Fig. 2A), so we divided the patients into two groups according to the mean miR-155 expression value (high or low). We found that there were no significantly differences of clinicopathological characteristics by miR-155 expression (Supplementary Table 2). Kaplan-Meier survival analysis showed that both overall survival time (*P* = 0.008) and disease-free survival time (*P* = 0.021) were significantly shorter in the high miR-155 expression group (Fig. 2B). Multivariate analysis following univariate analysis of the clinicopathological findings revealed that patients with high miR-155 expression had significantly poorer prognosis compared to patients with low miR-155 expression (Supplementary Table 3). Of the 45 patients, we analyzed the data from 15 patients who underwent GEM adjuvant therapy following R0 curative surgery in greater detail to gain insights into the clinical course of GEM resistance. The overall survival time (*P* = 0.006) was distinctly different in the high vs. low miR-155 expression groups (Fig. 2C).

In order to develop a clinical biomarker, the expression levels of exosomal miR-155 in plasma were evaluated in 23 patients whose pre-surgical plasma samples were available, and there was a significant correlation between the expression level of miR-155 in primary samples and in the corresponding exosomes (r^2^ = 0.71, *P* < 0.01) (Fig. 2D). When patients were divided into two groups according to the mean exosomal miR-155 expression value (high or low), exosomal miR-155 expression was a significant predictive factor for disease-free survival time (*P* = 0.017), but not for overall survival time (*P* = 0.200), although this may be because of the small number of patients (Supplementary Fig. 2B). These results demonstrated that high miR-155 expression predicts GEM resistance in the clinical setting and that exosomal miR-155 expression reflects PDAC miR-155 expression. Therefore, exosomal miR-155 expression may be a useful clinical prediction tool, but further investigation for verification of our findings is needed.

### High miR-155 expression induces GEM resistance via anti-apoptotic activity

**Figure 3:**
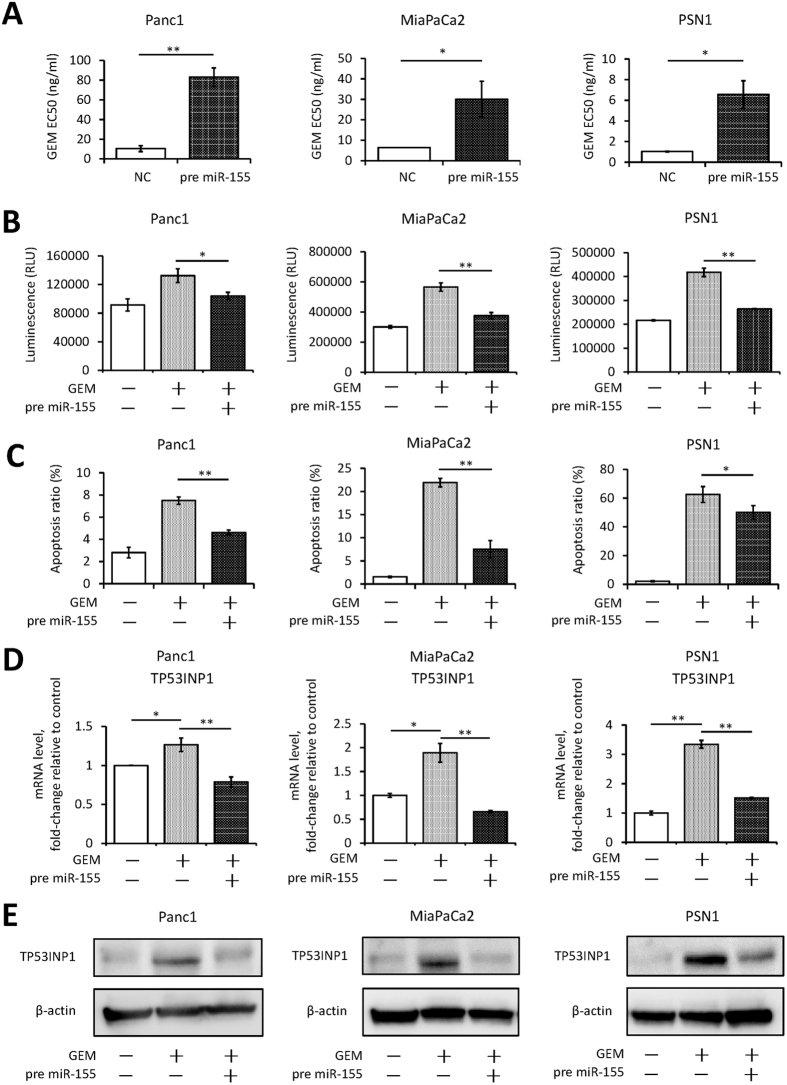
Transfecting cells with pre-miR-155 induces GEM resistance by enhancing anti-apoptotic activity. (**A**) The EC50 of GEM in each cell line as assessed by the MTT assay. Three different PDAC cell lines that overexpressed pre-miR-155 showed GEM resistance compared with cells transfected with a negative control oligonucleotide (NC). (**B** and **C**) Alterations in apoptosis in cell lines with pre-miR-155 or NC transfection after treatment with GEM for 72 hours. Caspase-3/7 activity is represented as the level of luminescence (**B**), and the ratio of apoptotic cells was determined using the Annexin V assay (**C**). (**D** and **E**) TP53INP1 expression in cells with per-miR-155 or NC transfection after treatment with GEM for 72 hours. mRNA expression was assessed using qRT-PCR (**D**). Immunoblot analysis was used to determine protein expression (**E**). Original images of immune blots on PVDF membrane are presented in Supplementary Fig. 4. (**A**–**E**) Concentrations of transfected pre-miR-155: Panc1, 10 nM; MiaPaCa2, 5 nM; and PSN1, 1 nM. (**B**–**E**) GEM concentrations: Panc1 and MiaPaCa2 cells, 50 ng/ml; PSN1, 10 ng/ml. Columns in (**A**–**D**) show values that are the averages of triplicate measurements; bars show SD values. Data are representative of three experiments. *P < 0.05; **P < 0.01.

To further investigate miR-155 activity, we overexpressed miR-155 in GEM-sensitive PDAC cells. Pre-miR-155 was transfected into parental PDAC cells (PDAC-Pt-OE). This transfection induced marked overexpression of mature miR-155 that continued for 72 hours (Supplementary Fig. 3A). Mature miR-155 was expressed spontaneously by each parental PDAC cell line, but the expression was significantly lower than in each Panc1-GR cell (Supplementary Fig. 3B). Overexpression of miR-155 did not affect cell viability (Supplementary Fig. 3C), indicating that miR-155 did not affect cell cycle regulation or cell proliferation per se. However, PDAC cells that overexpressed pre-miR-155 showed GEM resistance (Fig. 3A), suggesting that miR-155 affected the mechanisms involved in PDAC cell apoptosis. Two different apoptosis assays conducted in three PDAC cell lines showed that miR-155 transduction resulted in remarkably strong anti-apoptotic activity in PDAC cells (Fig. 3B and C). TP53INP1, a well-known target of miR-155, is a pro-apoptotic stress-induced p53 target gene^16^. It was previously reported that GEM treatment induced apoptosis with TP53INP1 increase^17^. Both protein and mRNA of TP53INP1 expression were increased by GEM treatment in control PDAC cells as previously reported, however, PDAC cells that overexpressed miR-155 did not show increased TP53INP1 expression even after GEM treatment (Fig. 3D and E). These results support the conclusion that high miR-155 expression is related to GEM resistance via an anti-apoptotic pathway.

### Increased miR-155 expression increases exosome secretion and the miR-155 content of exosomes, leading to GEM resistance

**Figure 4:**
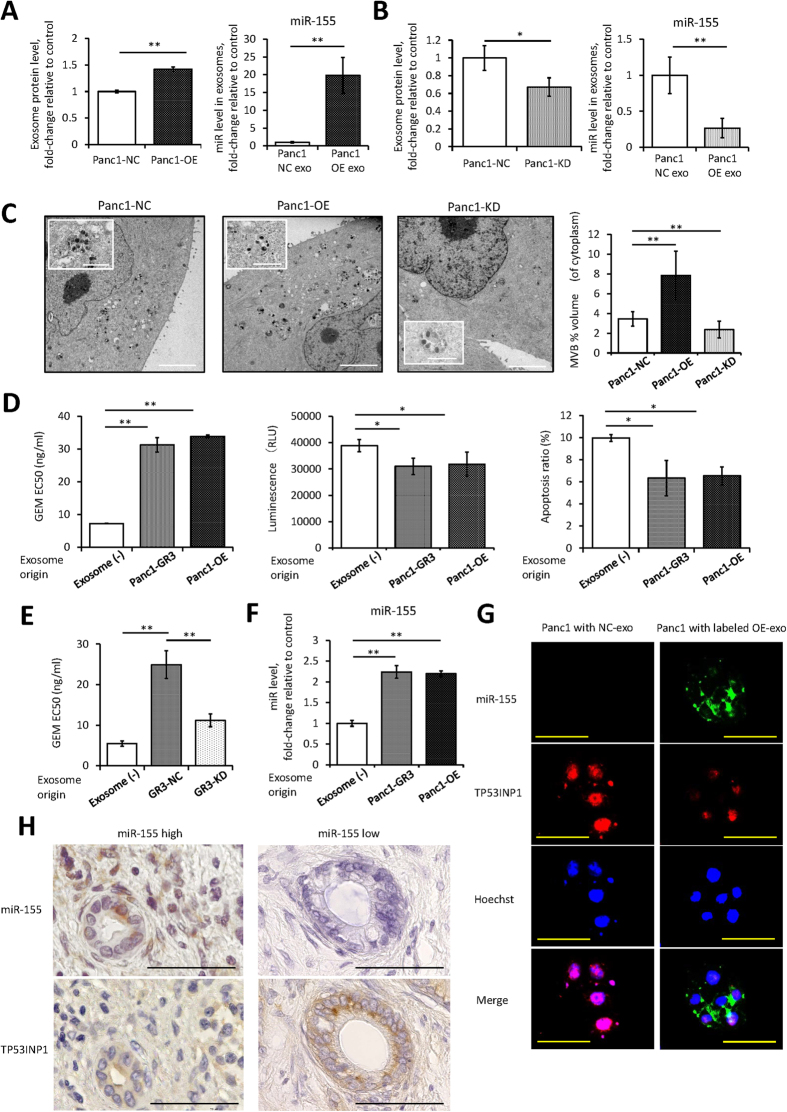
(**A**) The protein levels in exosomes isolated from Panc1 cells transfected with a negative control oligonucleotide (Panc1-NC), and cells transfected with pre-miR-155 (Panc1-OE) for 24 hours as assessed by Bradford method (left). The expression of miR-155 in each exosome (right). (**B**) The protein levels and the expression of miR-155 in exosomes isolated from Panc1-NC, and cells transfected with anti-miR-155 (Panc1-KD) for 48 hours. (**C**) Electron microscopy of Panc1-NC, Panc1-OE, or Panc1-KD. Representative pictures are shown of each cell (scale bar, 1 μm) as are close-up views of MVBs (scale bar, 100 nm). MVBs were quantified as the volume relative to the cytoplasm volume (right). (**D**) The EC50 of GEM in Pnac1-Pt cells that were treated with different types of exosomes for 48 hours was assessed by MTT assay (left). Apoptosis was assessed in Panc1-Pt cells that were treated with both GEM (50 ng/ml) and with isolated exosomes for 72 hours. Caspase-3/7 activity is represented as the level of luminescence (middle), and the ratio of apoptotic cells was determined using Annexin V assay (right). (**E**) Alterations in the GEM EC50 values in Pnac1-Pt cells after treatment with exosomes isolated from GR3-KD and GR3-NC. (**F**) The expression of miR-155 in Panc1-Pt cells after treatment with exosomes isolated from Panc1-GR3 or Panc1-OE. (**G**) The presence of Alexa 488-labeled miR-155 and TP53INP1 expression in recipient Panc1 cells treated with labeled OE-exo or with NC-exo. miR-155 or TP53INP1 are exhibited as green or red signal, respectively. The nuclei are stained with Hoechst (blue; scale bar, 100 μm). (**H**) The representative expression of miR-155 by *in situ* hybridization in pancreatic cancer cells with high or low expression of miR-155 from laser micro dissection (upper). The corresponding expression of TP53INP1 by immunohistochemistry at the same point in serial section specimen (lower) (scale bar, 50 μm). Columns in (**A**–**F**) show values that are the averages of triplicate measurements; bars show SD values. Data are representative of three experiments. *P < 0.05; **P < 0.01.

To investigate whether alterations in miR-155 expression affected exosome secretion volume or contents, exosomes were isolated from PDAC cells transfected with pre-miR-155 or anti-miR-155. Pre-miR-155 transfection increased exosome secretion and the expression level of miR-155 in the exosome (Fig. 4A); conversely, anti-miR-155 transfection decreased exosome secretion and the expression level of miR-155 in the exosome (Fig. 4B). To visualize these changes in cells, multivesicular bodies (MVBs) were observed by electron microscopy. We found that the MVB density in cells was increased by pre-miR-155 transfection and inhibited by anti-miR-155 transfection, indicating that miR-155 expression regulates exosome biogenesis and secretion (Fig. 4C).

To assess the role of exosomes in chemoresistance, GEM-sensitive Panc1 cells were treated with 50 ng/ml GEM along with a certain number of exosomes isolated from cells under each condition. The exosomes, which were derived from Panc1 cells that were overexpressing miR-155, strongly induced GEM resistance and had anti-apoptotic activity that was as strong as that of exosomes from GEM-resistant Panc1 cells (Fig. 4D). To validate the effect of the exosome from another PDAC cell line, the MiaPaCa2 cell line was employed. The exosomes, which were derived from MiaPaCa2 cells that were overexpressing miR-155, also induced GEM resistance and enhanced the anti-apoptotic activity for Panc1 and MiaPaCa2 (Supplementary Fig. 5A and B). Interestingly, exosomes from GEM-resistant cells transfected with anti-miR-155 attenuated the induction of GEM resistance, even though the same number of exosomes were added to the cells (Fig. 4E). Moreover, the expression of miR-155 by cells treated with exosomes from Panc1 cells transfected with anti-miR-155 was not significantly different from the expression of miR-155 by cells treated with control exosomes (Supplementary Fig. 5D), despite the significant escalation of miR-155 expression in cells treated with exosomes from Panc1 cells transfected with pre-miR-155 (Fig. 4F). These findings suggest that miR-155 not only increases exosome secretion but also that it changes the content of exosomes, including the miR-155 content, which can then lead to GEM resistance. To clarify the role of the exosome expressing miR-155, the exosome isolated from the cells transfected with Alexa 488-labeled miR-155 were added to Panc1 cells. The transition of Alexa 488-labeled miR-155 supported that exosomes played a role of transporter (Fig. 4F–G), moreover, the cells decrease TP53INP1 expression when the exosomes expressing miR-155 are endocytosed into the cells under GEM treatment (Fig. 4G and Supplementary Fig. 5E). To ascertain this serial reaction in clinical samples, we investigated the expression of miR-155 and TP53INP1 at the same point in the tissue, and as expected, the expression of TP53INP1 was inversely related to that of miR-155 (Fig. 4H).

### Blocking exosome delivery ameliorates miR-155-induced GEM resistance

**Figure 5:**
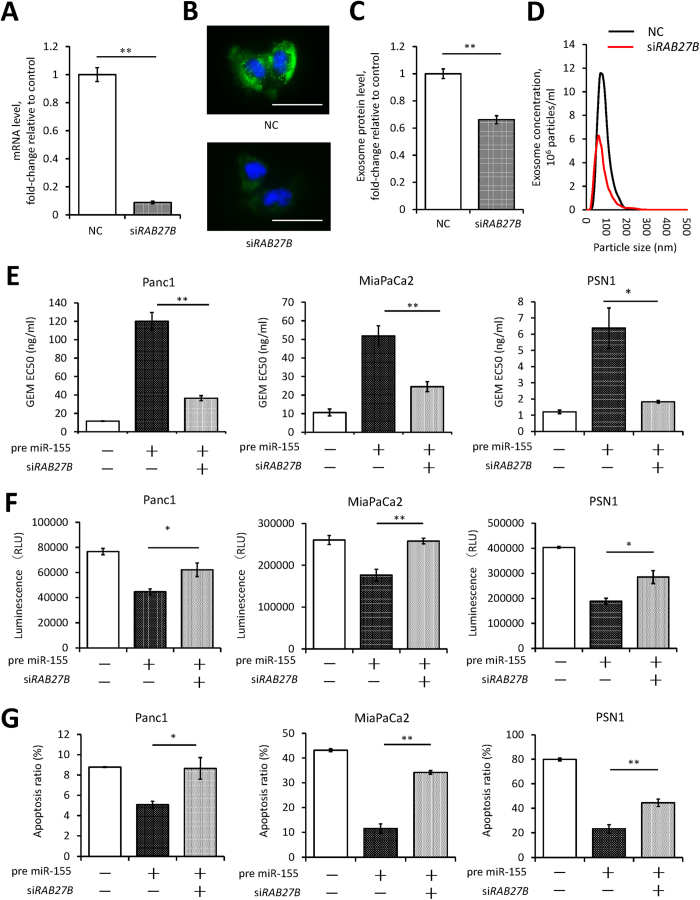
Inhibition of exosome synthesis attenuates the induction of GEM resistance by miR-155. (**A**) The expression of RAB27B in Panc1 cells transfected with a negative control oligonucleotide (NC) or with si*RAB27B* (90 nM siRNA). mRNA expression were assessed by qRT-PCR. (**B**) Immunocytochemistry shows RAB27B expression as a green signal in the cytoplasm of Panc1 cells. The nuclei were stained with Hoechst (blue; scale bar, 100 μm). (**C** and **D**) The amount of exosomes that was isolated from Panc1 cells after transfection with NC or with si*RAB27B* for 48 hours. The amount of exosomes was evaluated by the protein level by the Bradford method (**C**) or by the concentration of particles of a certain size (**D**). (**E**) Alterations of the EC50 of GEM in PDAC cells with si*RAB27B* or NC transfection. All PDAC cell lines showed ameliorated GEM resistance after transfection with si*RAB27B*, even cells transfected with pre-miR-155. (**F** and **G**) Alterations in apoptosis after 72 hours of GEM treatment in PDAC cells transfected with si*RAB27B* or with NC. Caspase-3/7 activity is represented as the level of luminescence (**F**), and the ratio of apoptotic cells was determined using the Annexin V assay (**G**). (**E**–**G**) Concentrations of transfected pre-miR-155: Panc1, 10 nM; MiaPaCa2, 5 nM; PSN1, 1 nM. Concentration of si*RAB27B* in the three cell lines, 90 nM). Concentration of GEM: Panc1 and MiaPaCa2, 50 μg/ml; and PSN1, 10 μg/ml. Columns in (**A**,**C**), and (**E**–**G**) show values that are the averages of triplicate measurements; bars show SD values. Data are representative of three experiments. *P < 0.05; **P < 0.01.

To clarify whether miR-155 by itself leads to GEM resistance or whether exosome delivery plays a dominant role, we used si*RAB27B* transfection to inhibit exosome secretion. After confirming that si*RAB27B* transfection efficiently downregulated RAB27B (Fig. 5A and B), we found that the number of exosomes was indeed reduced by RAB27B knockdown (Fig. 5C and D). Transfection with si*RAB27B* ameliorated the induction of GEM resistance in PDAC cells that were overexpressing miR-155 (Fig. 5E). Furthermore, RAB27B knockdown increased caspase-3/7 activity and the apoptotic cell ratio after GEM treatment, even in PDAC cells that were overexpressing miR-155 (Fig. 5F and G). These results suggest that miR-155 induces GEM resistance in PDAC cells not only due to its intrinsic functionality but also by increasing exosome secretion.

### Exosomes from cells overexpressing miR-155 induce GEM resistance in a murine xenograft model

**Figure 6:**
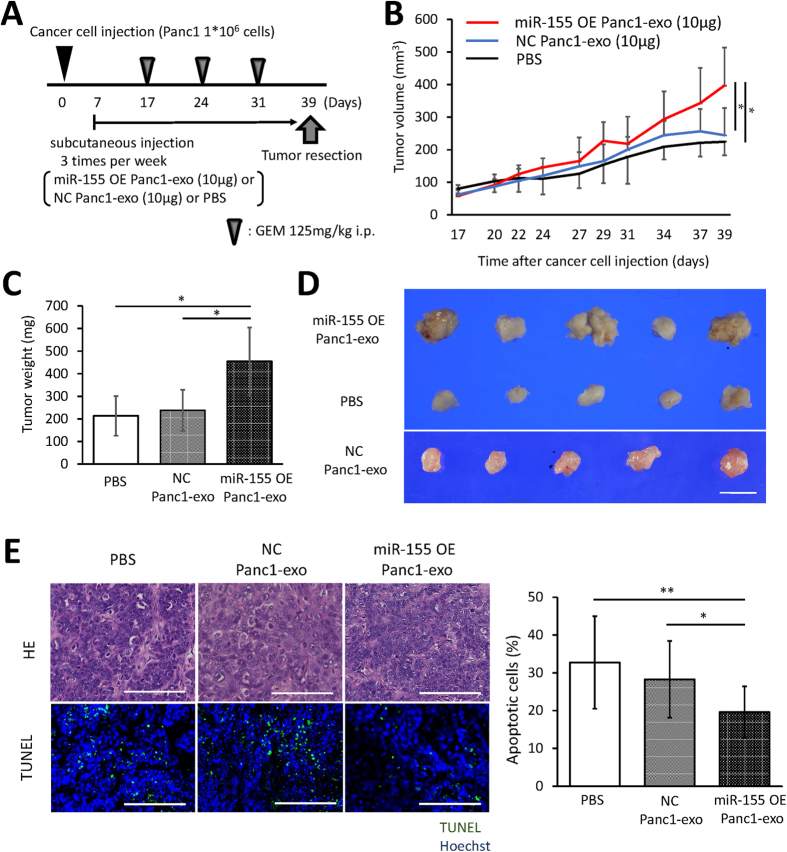
(**A**) The time course of the animal experiment. GEM was injected intraperitoneally when the tumor volume was 60–100 mm^3^. All mice were treated with exosomes isolated from Panc1 cells that were overexpressing miR-155 (miR-155 OE Panc1-exo) or negative control (NC Panc1-exo) or with PBS and were sacrificed at the same time. (**B**) The size of the subcutaneous tumors in NOD/SCID mice was measured at the time of each injection. Tumor volume was calculated based on the size of the tumor. (**C** and **D**) A comparison of the growth of tumors treated with miR-155 OE Panc1-exo or with NC Panc1-exo or with PBS. The tumors were resected from mice at the same time i.e. on day 39 after the xenograft. Tumor weight (**C**) and the actual tumors (**D**) are shown (scale bar, 1 cm). (**E**) A comparison of apoptotic cells in tissue from tumors treated with miR-155 OE Panc1-exo or with NC Panc1-exo or with PBS. Hematoxylin-eosin staining of the tumor specimens is shown in the top panels, and TUNEL staining of the same site on the sequential section is shown in the bottom panels. The TUNEL kit stains apoptotic cells green, while the nuclei are stained blue using Hoechst (scale bar, 100 μm). The right panel shows the rate of apoptosis as determined by counting the TUNEL-stained cells in the specimen. Columns in (**B** and **C**) show the average values in five samples; bars show SD values. Columns in (**E**) show the average values of five samples; each measurement was conducted in triplicate. *P < 0.05; **P < 0.01.

To confirm that exosomes from cells that overexpress miR-155 play a role in GEM resistance *in vivo*, we used a subcutaneous murine xenograft model and the experimental schema shown in Fig. 6A. Exosome treatment which was isolated from cells that overexpress miR-155 significantly attenuated the loss in tumor volume and weight caused by GEM treatment compared with exosome treatment which was isolated from cells that negative control transfection or PBS treatment (Fig. 6B–D). A TUNEL assay performed on resected tumors showed a significant decrease in apoptotic cells in tumors treated with exosomes from cells that overexpress miR-155 (Fig. 6E). These experiments demonstrated that exosomes secreted by cells that overexpress miR-155 can induce GEM resistance in tumor tissues *in vivo*.

## Discussion

This study had three important findings. First, long-term exposure to GEM increased miR-155 expression in PDAC cells. Second, the miR-155 expression level was positively related to exosome secretion that promoted GEM resistance, a finding that was validated in clinical samples and in *in vivo* experiments. Third, increasing the miR-155 level in PDAC cells while blocking exosome secretion did not induce GEM resistance. This study thus provides insights into the mechanisms underlying the development of GEM resistance in PDAC cells, showing that the positive feedback process involves increased exosome secretion following increased miR-155 expression.

Several miRNAs have been reported to be associated with drug resistance to GEM^18^,^19^,^20^,^21^,^22^,^23^, and miR-155 was found previously to be an oncogene in a wide variety of cancers^24^. In PDAC, increased miR-155 levels lead to downregulation of its target genes, which include *Sel*-*1*-*like*^25^, Mut L homologue 1^26^, and TP53INP1^16^. A meta-analysis suggested that overall survival was significantly shorter in patients with high miR-155 expression in their tumors^27^, similar to our findings in clinical samples. The majority of miR-155 target genes are related to anti-apoptosis or to tumorigenicity^16^. Our experiments indicate that the PDAC cells overexpressing miR-155 significantly developed anti-apoptotic activity under GEM treatment despite no difference in a stable state. Meanwhile, there were small differences of cell viability in PDAC cells with or without pre-miR-155 transduction. These findings suggest that miR-155 plays the dominant role in survival against GEM treatment. We also found that high miR-155 expression led to increased exosome secretion. Since exosomes can transfer functional miRNAs to recipient cells^28^,^29^,^30^, these results raised the possibility that GEM chemoresistance was due only to high intracellular miR-155 levels. Alternatively, exosome secretion might also be involved. To investigate these possibilities, we performed RAB27B knockdown to inhibit exosome secretion and found that this ameliorated the induction of GEM resistance even in cells that were overexpressing miR-155. These data suggested that the acquisition of chemoresistance required the exosome secretion pathway. Exosome secretion is known to be related to cell growth state (proliferation), density, and artificial intervention. However, there is no obvious difference between the backgrounds of Panc1-Pt and GEM-resistant cell lines, as indicated in Supplementary Fig. 1A; meanwhile, the number of exosomes in the supernatant of GEM-resistant cell line increased (Fig. 1B). These findings suggest that the increase of exosomes in the supernatant of GEM-resistant cell lines was not induced by these general reasons. To clarify the role of the exosome in GEM-resistant stable cell lines, the identification of the phenotype of GEM-resistant cell lines is necessary. We regarded high miR-155 expression as a feature of GEM-resistant stable cell lines, and high expression of miR-155 on exosomes would indicate exosome origin. The exosome derived from GEM-resistant cell lines showed high expression of miR-155 (Fig. 1G), and the cells showed increased anti-apoptotic activity with decreased TP53INP1 expression when the exosomes expressing miR-155 were endocytosed into the cells (Fig. 4D–G).

PDAC cells effectively evade chemotherapy by a number of different processes and strategies. Among these, researchers are increasingly focusing on exosomes that act as mediators of intercellular communication^9^. Exosomes originate by inward budding into MVBs^31^. Upon MVB fusion with the plasma membrane, the intraluminal vesicles are released into the extracellular space as exosomes. These processes are regulated by many genes, e.g. the RAB family of genes, including RAB27A/B, RAB11, and RAB35^32^,^33^,^34^. Several studies have reported that high RAB27B expression is significantly associated with poor overall survival in cancer patients, indicating that increasing exosome secretion worsens patient prognosis by contributing to chemoresistance^35^,^36^. Although all mRNA species can be targeted by several miRNAs, few studies have addressed the relationship between miRNA expression and MVB formation. We found that the miR-155 expression level was positively related to the formation of MVBs, which may suggest that miR-155 controls certain genes related to exosome synthesis. To consider how miR-155 controls exosome synthesis, we are now conducting experiments to test further hypothesis. As described above, RAB proteins positively controlled intracellular vesicle transport^37^, and the Tre2-Bub2-Cdc16 (TBC) domain-containing RAB-specific GTPase-activation proteins (TBC/RABGAPs) are key members of the family of RAB regulators^38^. Our new hypothesis is that one of the target genes of miR-155, conserved TBC/RABGAPs genes, act as negative regulators of RAB family genes, and miR-155 may increase exosome secretion through the decrease of these inhibitors; this is because the Target Scan prediction algorithm indicated that miR-155 could target conserved sites of TBC/RABGAPs genes. Further research is needed to better understand the underlying mechanisms.

In summary, this work demonstrates a novel role for miRNA in inducing exosome release that leads to GEM resistance in PDAC. Our results suggest that miR-155 effectively elicits GEM resistance in PDAC cells via a positive feedback process. In this process, an increase in cellular miR-155 expression has anti-apoptotic effects and also leads to exosome release by the cell. The exosomes then deliver chemoresistance-related substances, including miR-155, to other cancer cells, and the recipient cells subsequently develop chemoresistance and show an increase in exosome synthesis. This process represents a novel target in PDAC therapy that could be exploited in order to reduce or eliminate GEM resistance and to help improve the prognosis of PDAC patients.

## Materials and Methods

### Cell culture and GEM treatment

This study used three PDAC cell lines (Panc1, MiaPaCa2, and PSN1 cell lines). Panc1 was obtained from the ATCC, MiaPaCa2 was obtained from Japan Cancer Resource Bank (Toyko, Japan), and PSN1 was obtained from ECACC. The cells were cultured in Dulbecco’s modified Eagle’s medium supplemented with 10% fetal bovine serum (FBS) with antibiotics and incubated at 37 °C in a humidified incubator with 5% CO_2_. The FBS was first depleted of bovine exosomes by overnight ultracentrifugation at 120,000 × g followed by filtration (0.22-μm filters). We used three stable GEM-resistant cell clones established from Panc1 cells and named them Panc1-GR1, -GR3, and -GR4 cells as reported previously^18^. GEM was purchased from Eli Lilly Pharmaceuticals (Indianapolis, IN, USA).

### Exosome purification

Exosomes were isolated as described previously^39^. Briefly, conditioned medium was harvested 48 hours after cell seeding. After centrifugation at 3000 *g* to remove cellular debris, the supernatant was filtered through 0.22-μm PVDF filter (Millipore) to remove large vesicles. Exoquick Exosome Precipitation Solution (System Biosciences) was added to the filtered culture medium and mixed well. After refrigeration for 12 h, the mixture was centrifuged at 1500 g for 30 min. Exosome pellets were resuspended with PBS. The protein concentrations were measured by the Bradford method using a protein assay kit (Bio-Rad) as previously reported^40^. To confirm the contents of the purified exosome samples, we performed immunoblotting to detect tetraspanin CD63 (Supplementary Fig. 6A). The size distribution of the exosomes in the culture supernatants was evaluated with the NanoSight LM10 system using Nanoparticle Tracking Analysis (NTA) software v2.3 (NanoSight Ltd, Amesbury, UK). The mean vesicle size in the samples was 89 nm (Supplementary Fig. 6B), in accordance with previous reports^35^,^41^. Purified exosomes were added to the culture medium at a concentration of 50 μg/ml. For plasma samples, exosome isolation was performed using ExoQuick Plasma prep and the Exosome precipitation kit (System Biosciences).

### RNA extraction

Total RNA, including the small RNA fraction, was extracted from cells using Trizol reagent (Invitrogen)^42^. Exosomal RNA was isolated using the mirVana PARIS Kit (Ambion) as described previously^28^. Briefly, 250 μg of exosomes were diluted with 250 μl of PBS, and 1 μl of a 1 nM solution of cel-miR-39 was added to each aliquot as an external control for the following experiment. Samples were treated by phenol extraction and filtered through a cartridge according to the manufacturer’s protocol. To characterize the exosomal RNA, we tested the integrity of the RNA in the exosome fraction and specifically investigated the enrichment of small RNAs using a 2100 Bioanalyzer (Agilent Technologies) (Supplementary Fig. 6C).

### qRT-PCR to detect microRNA and messenger RNA expression

The reverse transcription (RT) reaction for microRNA was performed with the TaqMan MicroRNA RT Kit (Applied Biosystems, Foster City, CA, USA), and quantitative real-time polymerase chain reaction (qRT-PCR) was performed using TaqMan MicroRNA Assays (Applied Biosystems) and the ABI7900HT system (Applied Biosystems). The data were analyzed according to the comparative CT method^43^.

For messenger RNA, complementary DNA was synthesized from total RNA using the SuperScript First-Strand Synthesis System (Invitrogen). qRT-PCR was performed using specific oligonucleotide primers and the LightCycler 480 Real-Time PCR system (Roche Diagnostics, Mannheim, Germany). To detect the amplification products, LightCycler-DNA master SYBR green I (Roche Diagnostics) was used as described previously^44^. The PCR primers are shown in Supplementary Table 4.

### MiRNA microarray experiments

Microarray analysis of the purified RNAs, which were obtained from Panc1-Pt and Panc1-GR1, -GR3, and -GR4 cells, was performed by Toray microRNA microarray system. Labeled RNAs were hybridized onto 3D-Gene Human miRNA Oligo chips (v.17.0; Toray Industries, Tokyo, Japan). Fluorescent signals were scanned with the 3D-Gene Scanner (Toray Industries, Japan) and analyzed using 3D-Gene Extraction software (Toray Industries, Japan). Raw and processed data from this analysis are deposited in the Gene Expression Omnibus (GEO) repository (accession number: GSE80616).

### Growth inhibition assay and determination of cell viability

Growth inhibition was assessed using the 3-(4,5-dimethylthiazol-2-yl)-2,5-diphenyl tetrazolium bromide (MTT; Sigma-Aldrich Co.) assay as described previously^45^. In brief, cells were incubated for 72 hours under several concentrations of GEM, and then cell viability was evaluated by absorbance using MTT solution. The results were expressed as the percentage of absorbance relative to that of untreated controls.

### Apoptosis assay

To quantify the level of cellular apoptosis, both caspase-3/7 activation and flow cytometry analysis of Annexin V were performed as described previously^46^,^47^. Gene-transfected PDAC cells were exposed for 72 hours to GEM; specifically, Panc1, MiaPaCa2, and PSN1 cells were incubated in 50 ng/ml, 50 ng/ml, and 10 ng/ml of GEM, respectively. Caspase-3/7 activity was evaluated using the caspase-Glo^®^ 3/7 Assay Kit from Promega (Madison, WI, USA), and the relative luminescence (RLU) was measured by a GloMax^®^ Microplate Luminometer (Promega). Apoptotic cells that were stained with Annexin V-FITC (BioVision Research Products, Mountain View, CA, USA) or propidium iodide (PI, BioVision Research Products) were counted by flow cytometry using the BD FACS Canto™ II system (BD Biosciences).

### Electron microscopy

Electron microscopy (Hitachi H-7650, Hitachi, Tokyo, Japan) was used to visualize multivesicular bodies (MVBs). Cells were fixed with Quetol-812 (Nissin EM, Tokyo, Japan) and cut into ultrathin (80-nm) sections using the Reichert-Jung Ultracut E (Reichert, Vienna, Austria). To quantify the MVBs, the volume density of MVBs was assessed in the cytoplasm of ten randomly chosen cells using ImageJ software (NIH, http://rsb.info.nih.gov/ij/).

### Transfection

All genes were transfected into cells using Lipofectamine RNAiMAX transfection reagent (Invitrogen)^19^. The precursor oligonucleotide of hsa-miR-155 (pre-miR-155), the antisense oligonucleotide inhibitor of hsa-miR-155 (anti-miR-155), and their scrambled oligonucleotides were obtained from Ambion Inc. (Austin, TX, USA). To visualize the transport of artificial microRNA, we used Alexa 488-conjugated mature miR-155 as follows: 5′-Alexa488 ssH amino linker UUAAUGCUAAUCGUGAUAGGGGU-3′; complementary: 5′-ACCCCUAUCACGAUUAGCAUUAA-3′ (Gene Design, Osaka, Japan). Small interfering RNA (siRNA) targeting *RAB27B* and a negative control oligonucleotide were purchased from Dharmacon (Thermo Fisher Scientific, Waltham, MA, USA) to inhibit exosome secretion. RAB27B GTPases were found previously to regulate the secretion of secretory exosomes^32^,^35^,^48^. Each gene and the corresponding negative control oligonucleotide were transfected.

### Protein detection

Immunoblotting or immunocytochemistry was performed to evaluate protein expression. For immunoblotting analysis, cellular and exosomal proteins were harvested with RIPA buffer^18^,^19^, and divided into aliquots that had a protein concentration of 1.0 μg/μl. Immunoblotting was performed using antibodies that detect CD63 (Santa Cruz), TP53INP1 (Santa Cruz), and ACTB (Sigma-Aldrich)^49^,^50^,^51^. Immunocytochemistry to detect RAB27B (Santa Cruz) and TP53INP1 was conducted on cells as described previously^52^. Antibody sources and dilutions are shown in Supplementary Table 4.

### Immunohistochemistry

Tissue samples of pancreatic cancer were obtained with institutional review board approval. Pancreatic cancer specimens were fixed in 4% paraformaldehyde for 48 hours, embedded in paraffin, and sectioned into 4.0-μm slices. Paraformaldehyde-fixed, paraffin-embedded pancreatic tissue sections were deparaffinized, hydrated, and incubated overnight at 4 °C with an anti-p53 DINP1 (1:500) from Abcam as the primary antibody. Bound antibodies were detected with biotin-conjugated secondary antibodies and diaminobenzidine (Vector Laboratories, Burlingame, CA) as a substrate, and the tissue slices were counterstained with hematoxylin.

### *In situ* hybridization

Super Sensitive One-Step Polymer-HRP ISH Detection System (Biogenex, Fremont, CA) was used for ISH in the present study according to the manufacturer’s protocol. Briefly, the ISH analysis was performed on 4.0-μm thick sections. Section were pre-digested with nucleic acid retrieval at 85 °C for 2 minutes and at 98 °C for 20 minutes, and pre-hybridized at 37 °C for 30 minutes, and hybridized with has-miR-155 fluorescenated oligo probe (BioGenex) at 42 °C for 2 hours. After washes, the sections were blocked with blocking buffer. For signal detection, anti-fluorescein antibody was used as primary antibody. Bound antibodies were detected with horse radish peroxidase-conjugated secondary antibodies and diaminobenzidine, and the tissue slices were counterstained with hematoxylin.

### Clinical samples

All patients provided written informed consent before inclusion in the study, and the study protocol was approved by the Human Ethics Review Committee of the Graduate School of Medicine, Osaka University (approval number 15319), which adhered to the guidelines of the Declaration of Helsinki. We analyzed samples from 45 consecutive PDAC patients who underwent histologically curative resection (R0) between March 2007 and August 2015 at Osaka University Hospital. To be eligible for inclusion, the patient must have received adjuvant chemotherapy or post-recurrence chemotherapy but not preoperative chemotherapy. Laser capture microdissection (LMD7000, Leica Microsystems GmbH, Wetzlar, Germany) was performed on resected specimens from these patients in order to collect epithelial adenocarcinoma lesions that were distinct from the surrounding stroma. Pre-surgical plasma samples were available from 23 of the 45 patients. The plasma levels of exosomal miR-155 were adjusted for body surface area.

### *In vivo* experiments

This study was approved by the Animal Experiments Committee, Osaka University (approval number, 25-045-006). It was performed in accordance with the National Institutes of Health guidelines for the use of experimental animals. Six-week-old female non-obese mice with diabetes/severe combined immunodeficiency were purchased from CLEA Japan and maintained in a pathogen-free environment. For the xenografts, Panc1 cells (1 × 10^6^) were subcutaneously transplanted in 100 μl PBS/Matrigel (BD Biosciences)^18^. Three times a week, either PBS or exosomes (10 μg) isolated from the supernatant of culture media from Panc1 cells overexpressing miR-155 or negative control were injected into a site adjacent to each tumor. The mice were administered GEM (125 mg/kg) intraperitoneally three times on days 17, 24, and 31 after xenograft transplantation. Subcutaneous tumor volume was calculated as follows: (greatest diameter) × (shortest diameter)^2^ × 0.5. GEM therapy was initiated when the tumor volume was 60–100 mm^3^. Mice were euthanized on day 39. The tumor tissue sections were stained with hematoxylin and eosin; alternatively, a TUNEL assay was performed on the sections using an *in situ* cell death detection kit (Roche Diagnostics).

### Statistical analysis

The clinicopathological parameters were compared using Fisher’s exact test, and continuous variables were compared using the Student’s t-test. The survival curves were computed using the Kaplan-Meier method. Cox’s regression was performed for multivariate survival analysis to determine the main independent risk factors for overall survival or disease-free survival. Correlations were determined using Pearson’s correlation. The level of significance was set at *P* ≤ 0.01 or *P* ≤ 0.05. All statistical analyses were performed with the IBM SPSS 20.0 software package (IBM Japan, Tokyo, Japan).

## Additional Information

**How to cite this article:** Mikamori, M. *et al*. MicroRNA-155 Controls Exosome Synthesis and Promotes Gemcitabine Resistance in Pancreatic Ductal Adenocarcinoma. *Sci. Rep.* **7**, 42339; doi: 10.1038/srep42339 (2017).

**Publisher's note:** Springer Nature remains neutral with regard to jurisdictional claims in published maps and institutional affiliations.

## Supplementary Material

Supplementary Information
