# Supplementary material for: MicroRNA-155 Controls Exosome Synthesis and Promotes Gemcitabine Resistance in Pancreatic Ductal Adenocarcinoma
Source: Sci Rep. 2017 Feb 15;7:42339. doi: 10.1038/srep42339 (PMC5309735; doi:10.1038/srep42339)
Supplement: Supplementary Information [file srep42339-s1.pdf]

Title page

Title: MicroRNA-155 Controls Exosome Synthesis and Promotes Gemcitabine Resistance in Pancreatic Ductal Adenocarcinoma

Authors

Manabu Mikamori,[1] Daisaku Yamada,[1] Hidetoshi Eguchi,[1] Shinichiro Hasegawa,[1] Tomoya Kishimoto,[1] Yoshito Tomimaru,[1] Tadafumi Asaoka,[1] Takehiro Noda,[1] Hiroshi Wada,[1] Koichi Kawamoto,[1] Kunihito Gotoh,[1] Yutaka Takeda,[1,2] Masahiro Tanemura,[1,3] Masaki Mori,[1] Yuichiro Doki,[1]

Affiliation

1. Department of Gastroenterological Surgery, Graduate School of Medicine, Osaka University, Yamadaoka 2-2, Suita, Osaka, Japan, 565-0871

2. Department of Surgery, Kansai Rosai Hospital,

Inabasou 3-1-69, Amagasaki, Hyogo, Japan, 660-8511

3. Department of Surgery, Osaka Police Hospital,

Tennoji-ku Kitayamacho 10-31, Osaka, Japan, 543-0035

Corresponding Author:

Hidetoshi Eguchi MD, PhD

Address: 2-2 Yamadaoka, Suita, Osaka, Japan, 565-0871

Tel: +81-6-6879-3251, Fax: +81-6-6879-3259

E-mail: heguchi@gesurg.med.osaka-u.ac.jp

Supplementary Figure 1

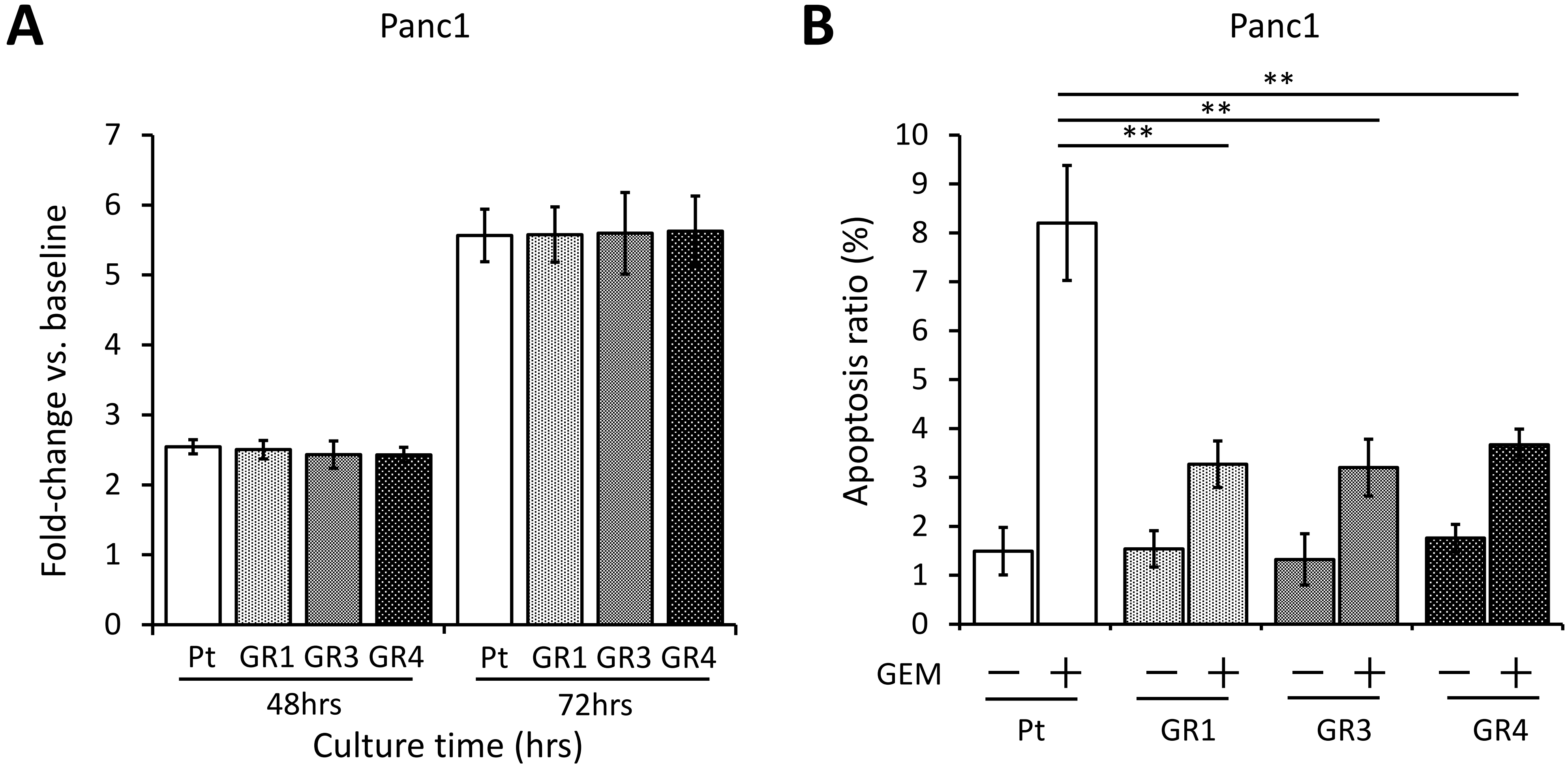

Supplementary Figure 1.
(**A**) Cellular viability after cell seeding is expressed as the fold-change relative to baseline and was calculated using the MTT assay in parental Panc1 (Panc1-Pt) cells and in GEM-resistant Panc1 cells (Panc1-GR). (**B**) Alterations in apoptosis in Panc1-Pt and Panc1-GRs cells after treatment with GEM for 72 hours. The ratio of apoptotic cells was determined using the Annexin V assay. Columns in A and B show values that are the average of triplicate measurements; bars show SD values. Data are representative of three experiments. **, *P*<0.01.

Supplementary Figure 2

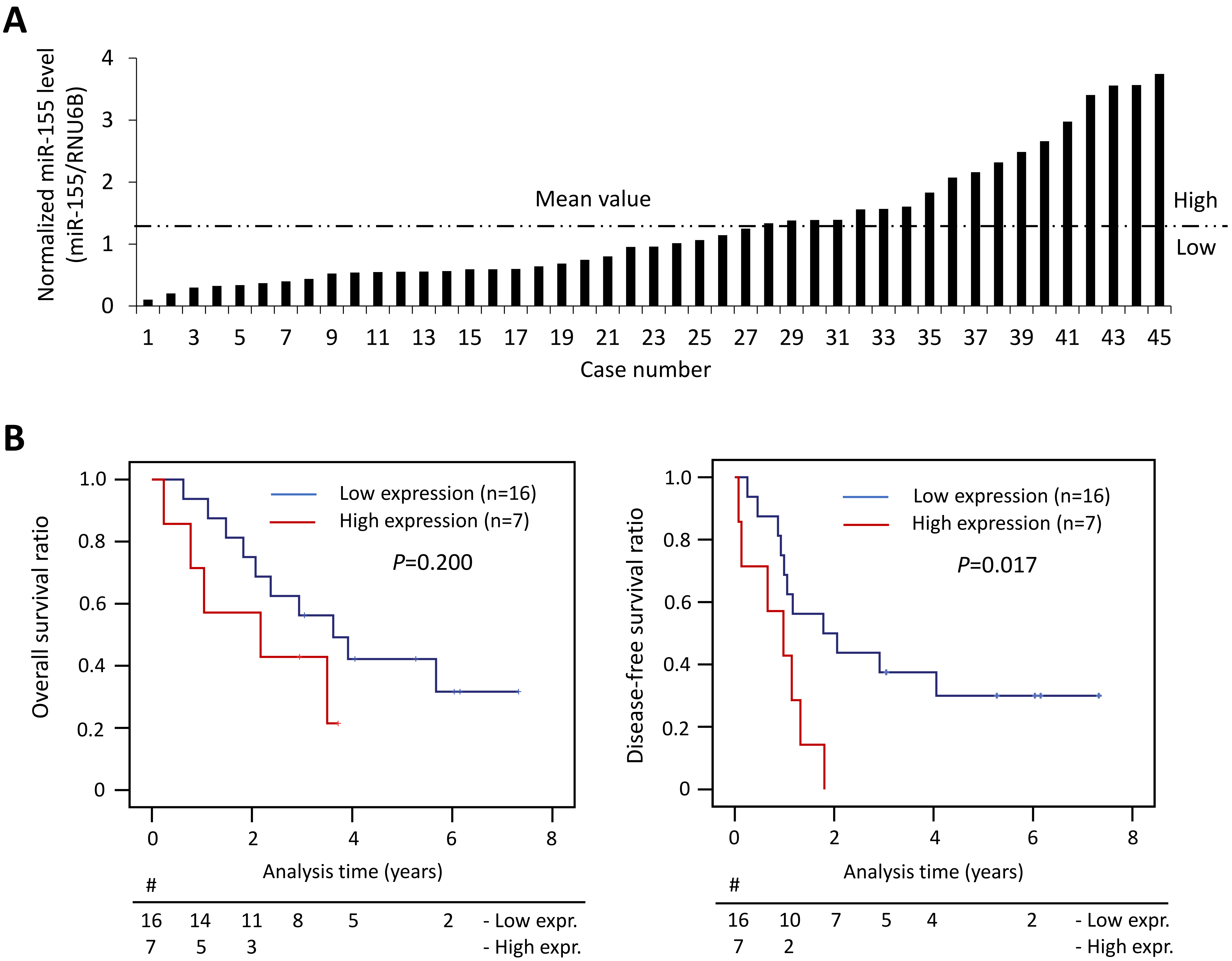

Supplementary Figure 2.
(**A**) Laser capture microdissection (LCM) was performed to collect material from tissue resected from 45 patients. Real-time qRT-PCR was used to assess the miR-155 expression level in each sample. The relative miRNA expression was quantified using the comparative CT method (2-ΔCT). The mean value of the miR-155 expression level in these samples was used as a cut-off value to divide the patients into two groups (high or low miR-155 expression). (**B**) Kaplan-Meier plots of overall survival (left) and disease-free survival (right) of 23 patients according to the expression of miR-155 in their plasma exosomes (*P* value, log-rank test).

Supplementary Figure 3

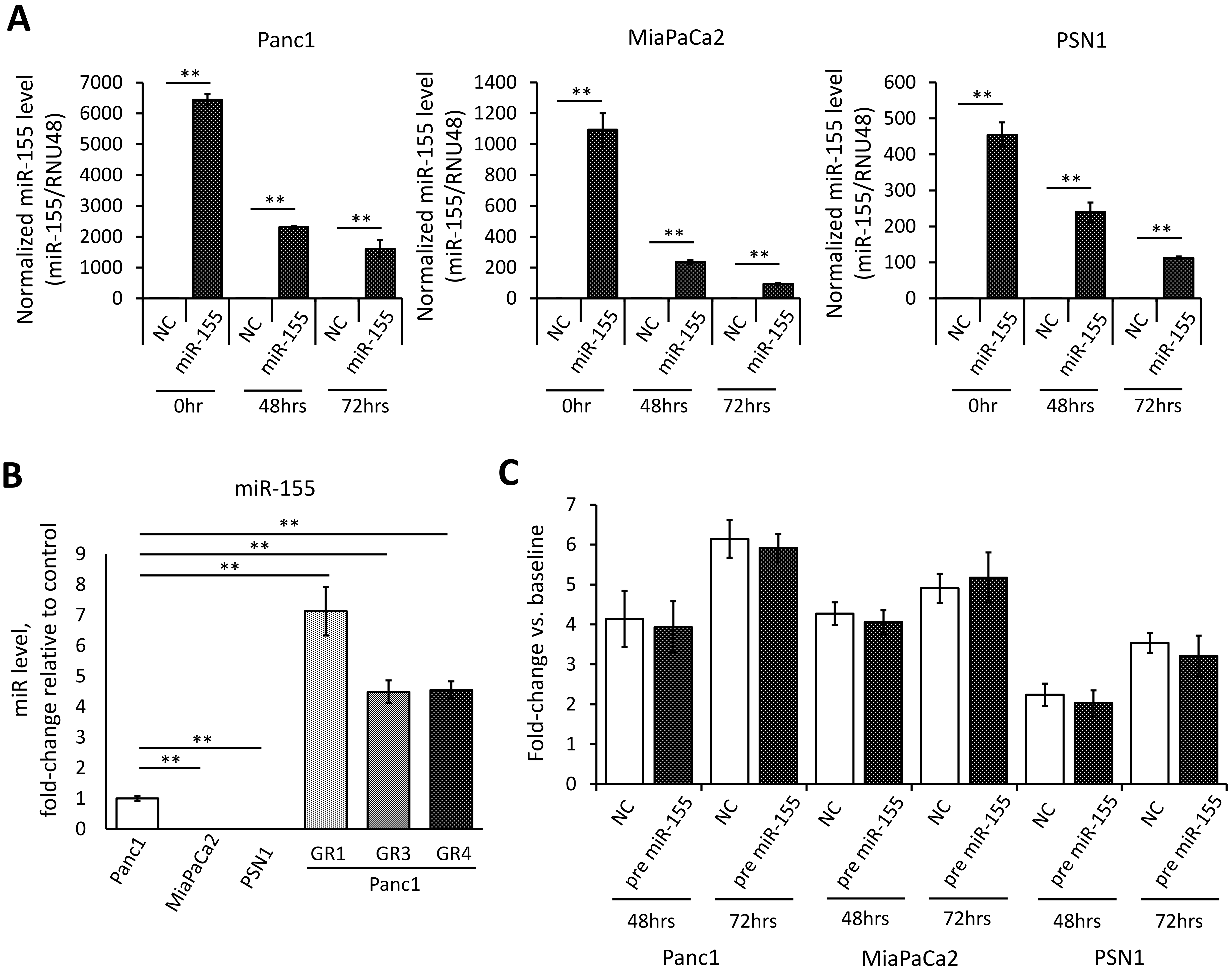

Supplementary Figure 3.

(**A**) The expression of miR-155 in PDAC cells transfected with pre-miR-155 or negative control oligonucleotides (NC) was detected by qRT-PCR. Concentrations of transfected pre-miR-155: Panc1, 10 nM; MiaPaCa2, 5 nM; and PSN1, 1 nM. (**B**) qRT-PCR was used to detect the expression of mature miR-155 in each PDAC cell line and in Panc1-GR cells. (**C**) The viability of each PDAC cell line transfected with pre-miR-155 or NC after seeding, as calculated by the MTT assay; viability is expressed as the fold-change compared to baseline. Columns in A–C show the averages of triplicate measurements; bars show SD values. Data are representative of three experiments. **, *P*<0.01.

Supplementary Figure 4

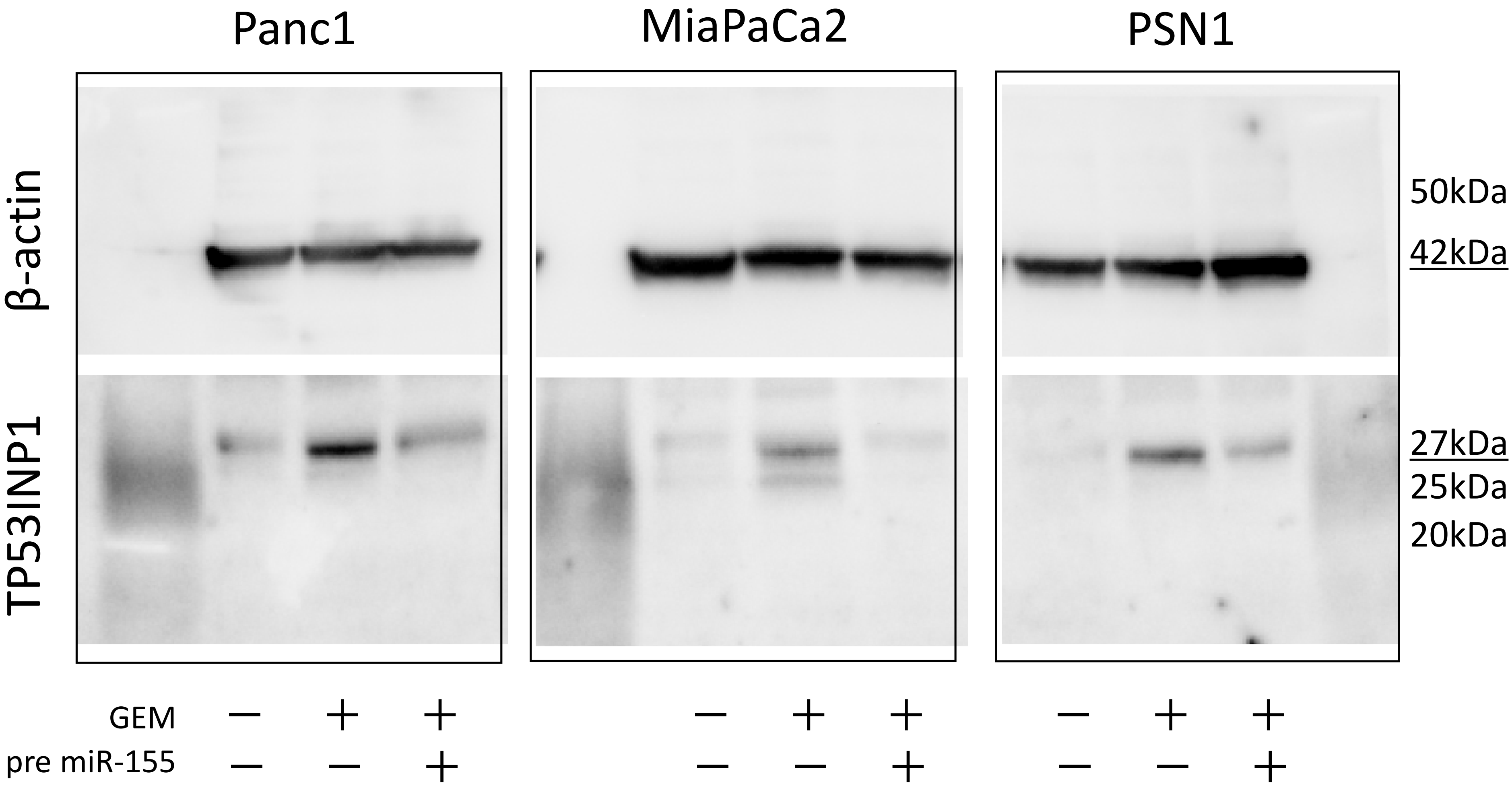

Supplementary Figure 4.

Original images of immune blots on PVDF membrane. A membrane were cut to 6 parts at 35 kDa, and each part of membrane were separately treated from the procedure of incubation with primary antibody (TP53INP1: 27kDa, β-actin: 42kDa). Modified image to show each band more clearly were depicted in figure 3E.

Supplementary Figure 5

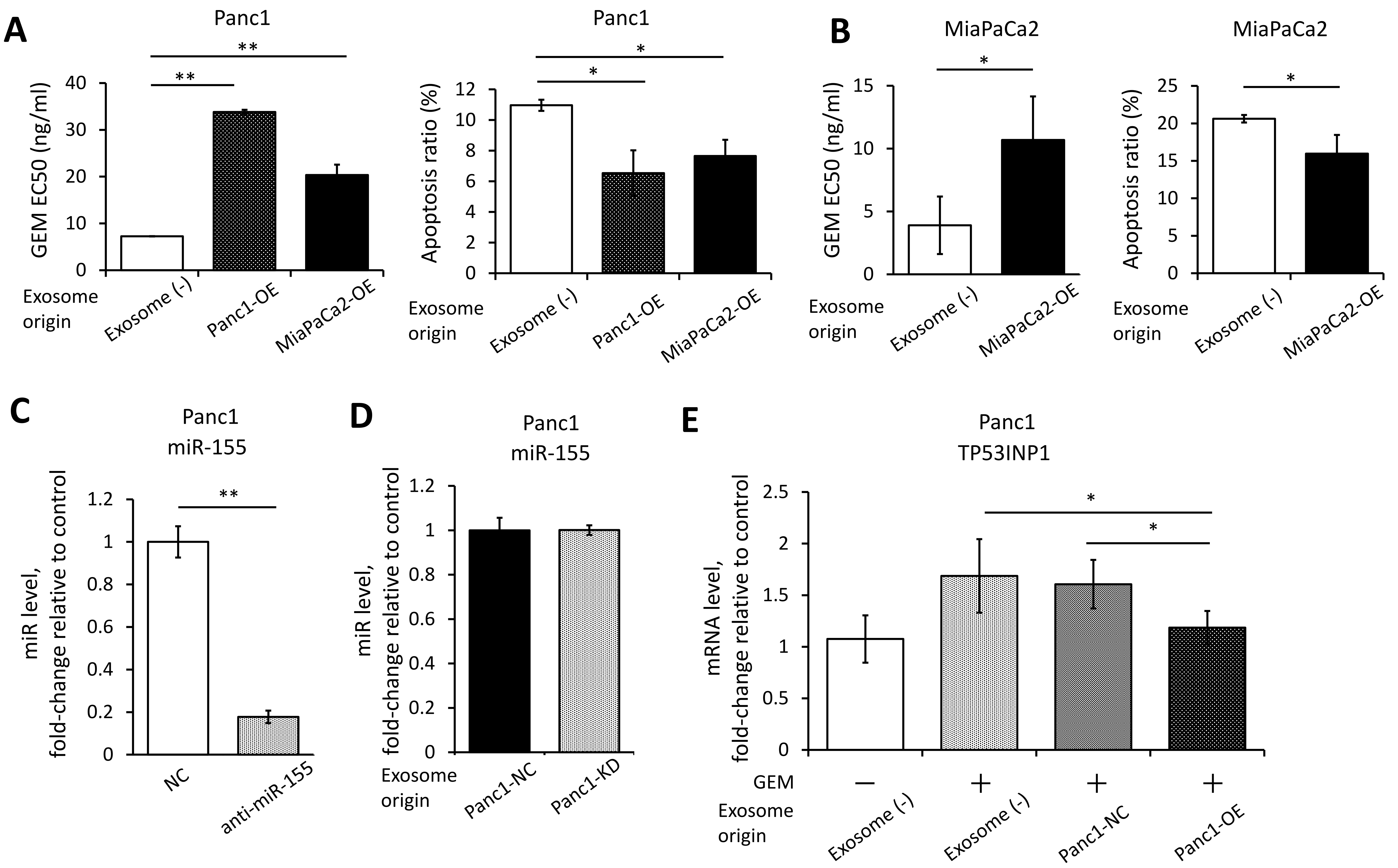

Supplementary Figure 5.

(**A**) The EC50 of GEM in Pnac1-Pt cells that were treated with exosomes isolated from PDAC cells transfected with pre-miR-155 (Panc1-OE, MiaPaCa2-OE) for 48 hours was assessed by the MTT assay (left). Apoptosis ratio was assessed in Panc1-Pt cells after GEM (50 ng/ml) treatment with or without isolated exosomes for 72 hours. The ratio of apoptotic cells was determined using the Annexin V assay (right). (**B**) The EC50 of GEM in MiaPaCa2-Pt cells that were treated with exosomes isolated from MiaPaCa2-OE for 48 hours was assessed by the MTT assay (left). Apoptosis ratio was assessed in MiaPaCa2-Pt cells after GEM (50 ng/ml) treatment with or without isolated exosomes for 72 hours. The ratio of apoptotic cells was determined using the Annexin V assay (right). (**C**) The expression levels of miR-155 in Panc1 cells transfected with anti-miR-155 (50 nM) or with negative control oligonucleotides (NC) as determined by qRT-PCR. (**D**) qRT-PCR analysis shows the level of intracellular copies of miR-155 in recipient Panc1 cells that were treated for 48 hours with exosomes that were isolated from Panc1 cells transfected with anti-miR-155 (Panc1-KD). (**E**) TP53INP1 expression in Panc1-Pt cells after GEM/PBS treatment with or without the addition of exosomes isolated from Panc1-NC or Panc1-OE for 72 hours. mRNA expression was assessed using qRT-PCR. Columns in A –E show values that are the average of triplicate measurements; bars show SD values. Data are representative of three experiments. NS, not significant. *, $P<0.05$; **, $P<0.01$.

Supplementary Figure 6

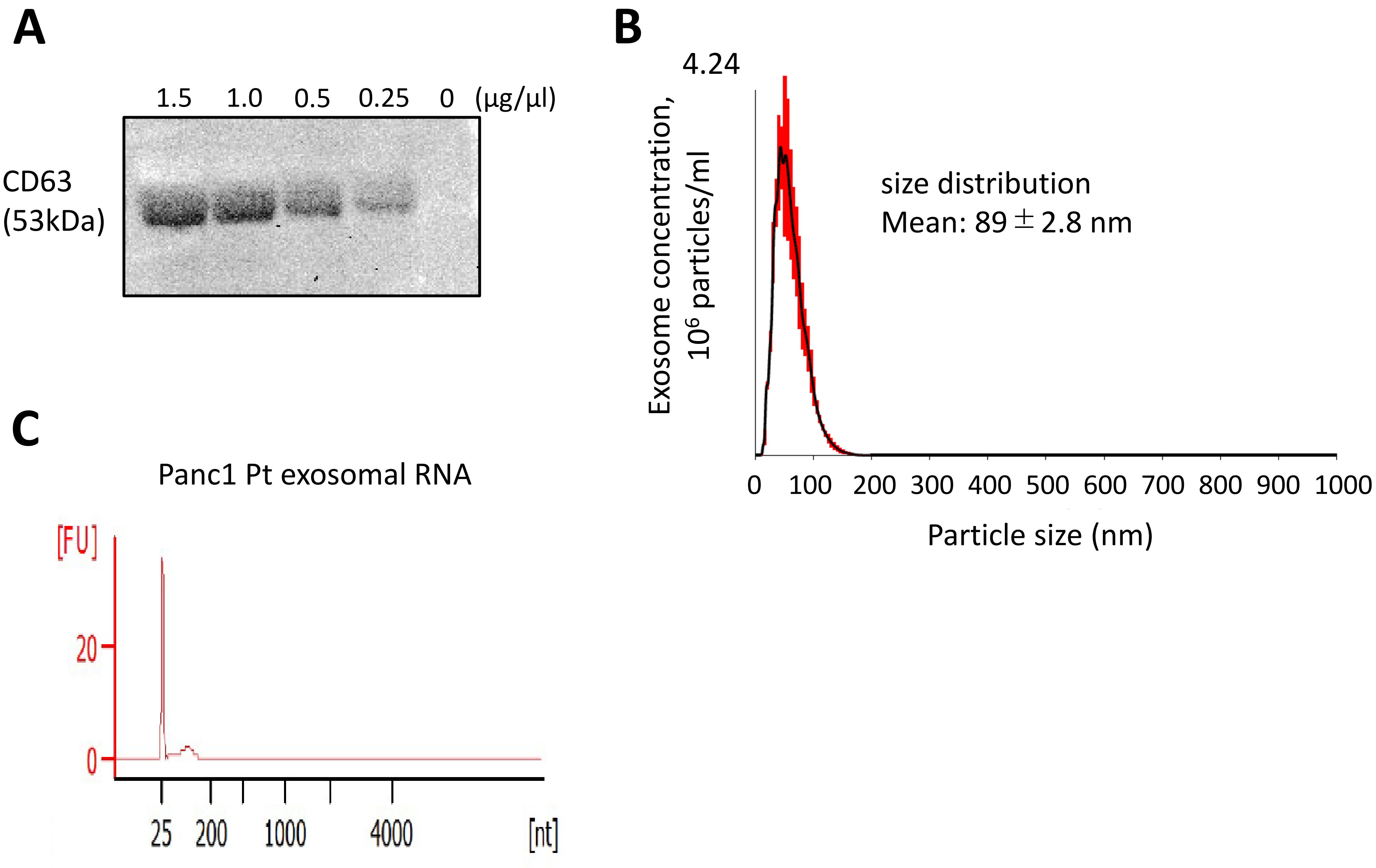

Supplementary Figure 6

(**A**) Expression of CD63, an exosomal marker, in exosomes isolated from Panc1 media supernatant. The lanes are arranged according to the amount of protein level. (**B**) The size distribution of exosomes secreted by Panc1 cells as determined by the NanoSight LM10 system. (**C**) Total RNA isolated from the exosome fraction was detected using the Bioanalyzer 2100. The data show the amount of total RNA and the size distribution of the isolated RNA. The columns in B show the averages of triplicate measurements; bars show SDs. Data are representative of three experiments.

Supplementary Table 1. Candidate miRNAs that showed expression levels that were altered more than 2.0-fold or less than 0.5-fold in Panc1-GR cells relative to Panc1-Pt cells

GR/Pt >2.0-fold		GR/Pt <0.5-fold	
hsa-miR-4521	7.21	hsa-miR-3620	0.34
hsa-miR-29b-1*	5.84	hsa-miR-323-5p	0.38
hsa-miR-362-3p	5.06		
hsa-miR-665	3.71		
hsa-miR-4711-3p	3.64		
hsa-miR-503	3.61		
hsa-miR-19b-1*	3.55		
hsa-miR-4721	3.44		
hsa-miR-424	3.34		
hsa-miR-421	3.29		
hsa-miR-1202	3.28		
hsa-miR-3130-5p	3.27		
hsa-miR-155	3.20		
hsa-miR-181d	3.12		
hsa-miR-193a-3p	3.11		
hsa-miR-1236	2.87		
hsa-miR-301b	2.80		
hsa-miR-4732-5p	2.76		
hsa-miR-4685-3p	2.76		
hsa-miR-3184	2.76		
hsa-miR-720	2.66		
hsa-miR-887	2.65		
hsa-miR-658	2.61		
hsa-miR-188-5p	2.61		
hsa-miR-1913	2.56		
hsa-miR-1224-3p	2.54		
hsa-miR-1280	2.46		
hsa-miR-4710	2.45		
hsa-miR-330-5p	2.41		
hsa-miR-1285	2.37		

Supplemental Table 2. The clinicopathological characteristics of the 45 patients who underwent histologically curative resection without any preoperative treatment and received adjuvant chemotherapy or post recurrence chemotherapy preoperative therapy

	miR-155 (low expression) n=27	miR-155 (high expression) n=18	*P*-value
Age (years)	70.0 ± 8.1	68.7 ± 9.9	0.64
Sex (male / female)	14 / 13	11 / 7	0.76
Histopathological type (well or moderate / poor)	24 / 3	17 / 1	0.64
Tumor size (mm)	27.9 ± 15.8	29.0 ± 13.0	0.81
Tumor location (head / body or tail)	14 / 13	11 / 7	0.76
Pathological depth of invasion, pT (T1 / T2 / T3)	3 / 4 / 20	0 / 0 / 18	0.06
Pathological lymph node metastasis, pN (negative / positive)	16 / 11	5 / 13	0.07
Pathological stage (IA / IB / IIA / IIB)	3 / 4 / 9 / 11	0 / 0 / 5 / 13	0.08

Abbreviations: poor, poorly differentiated; well, well differentiated

Supplementary Table 3. Univariate and multivariate analysis of predictive factors of overall survival in 45 patients with pancreatic ductal adenocarcinoma

	Univariate	Multivariate		
	P-value	Hazard ratio	95%CI	*P*-value
Age (years) (≥72 / <71: median)	0.09	1.96	0.83–4.64	0.12
Sex (male / female)	0.65			
Histopathological type (well or moderate / poor)	0.74			
Tumor size (mm) (≥25 mm / <25 mm: median)	0.50			
Tumor location (head / body or tail)	0.69			
Pathological depth of invasion pT (T1 or T2 / T3)	0.03			
Pathological lymph node metastasis pN (negative / positive)	0.01			
Pathological stage (IA or IB / IIA or IIB)	0.03	2.60	0.69–9.76	0.15
miR-155 expression (average)	0.01	2.63	1.07–6.46	0.04

Abbreviations: poor, poorly differentiated; well, well differentiated

Supplementary Table 4. Primers used for qRT-PCR and antibodies used for immunoblotting and immunocytochemistry.

Gene-specific primers	Sequences (5′ to 3′)
TP53INP1 forward	GCACCCTTCAGTCTTTTCCTGTT
TP53INP1 reverse	GGAGAAAGCAGGAATCACTTGTA
RAB27B forward	TGGATGAGCCAACTGCAAGC
RAB27B reverse	GCCGTTCATTGACTTCCCTCTCG
ACTB forward	TTAAGGAGAAGCTGTGCTACG
ACTB reverse	GTTGAAGGTAGTTTCGTGGAT

Antibody	Source	Dilution	Purchased from
Anti-CD63 (MX-49.129.5)	Mouse monoclonal	1:200	Santa Cruz Biotechnology
Anti-TP53INP1 (H-110)	Rabbit polyclonal	1:200	Santa Cruz Biotechnology
Anti-Rab27b (C-20)	Goat polyclonal	1:200	Santa Cruz Biotechnology
Anti-β-actin antibody	Rabbit monoclonal	1:1000	Sigma-Aldrich
